# Generation of two induced pluripotent stem cell (iPSC) lines from an ALS patient with simultaneous mutations in KIF5A and MATR3 genes

**DOI:** 10.1016/j.scr.2020.102141

**Published:** 2020-12-24

**Authors:** David X. Medina, Ashley Boehringer, Marissa Dominick, Ileana Lorenzini, Sara Saez-Atienzar, Erik P. Pioro, Rita Sattler, Bryan Traynor, Robert Bowser

**Affiliations:** a Department of Neurobiology, Gregory W Fulton ALS Research Center, Barrow Neurological Institute, 350 W Thomas Road, Phoenix, AZ 85013, USA; b Neuromuscular Diseases Research Section, Laboratory of Neurogenetics, NIA, NIH, 35 Convent Drive, Room 1A-213, Bethesda, MD 20892, USA; c Section of ALS & Related Disorders, Department of Neurology, S90, Neurological Institute, Cleveland Clinic, 9500 Euclid Ave, Cleveland, OH 44195, USA; d Department of Neurology, Johns Hopkins University, Baltimore, MD 21287, USA

## Abstract

Fibroblasts from an amyotrophic lateral sclerosis patient with simultaneous mutations in the MATR3 gene and KIF5A gene were isolated and reprogrammed into induced pluripotent stem cells via a non-integrating Sendai viral vector. The generated iPSC clones demonstrated normal karyotype, expression of pluripotency markers, and the capacity to differentiate into three germ layers. The unique presence of two simultaneous mutations in ALS-associated genes represent a novel tool for the study of ALS disease mechanisms.

## 1. Resource utility

Recently, new mutations in genes for Matrin 3 (MATR3) and Kinesin Family Member 5A (KIF5A) were linked to familial amyotrophic lateral sclerosis (ALS). We have generated two new iPSC lines from a patient that has simultaneous mutations in both the MATR3 (F115C missense mutation) and KIF5A (intronic mutation) genes.Table 1.Table 2.

## 2. Resource details

Amyotrophic lateral sclerosis (ALS) is a fatal neurodegenerative disease resulting from the loss of upper and lower motor neurons leading to progressive paralysis and eventual death typically between 2 and 5 years after diagnosis. The need for novel models to study disease mechanisms is essential for the development of improved treatments. Here we report novel, patient-derived iPSC lines which were derived from a patient that was identified to have simultaneous mutations in two ALS related genes (MATR3 and KIF5A). This cell line provides an opportunity to study individual and interacting effects of multiple mutations.

**Fig. 1.**
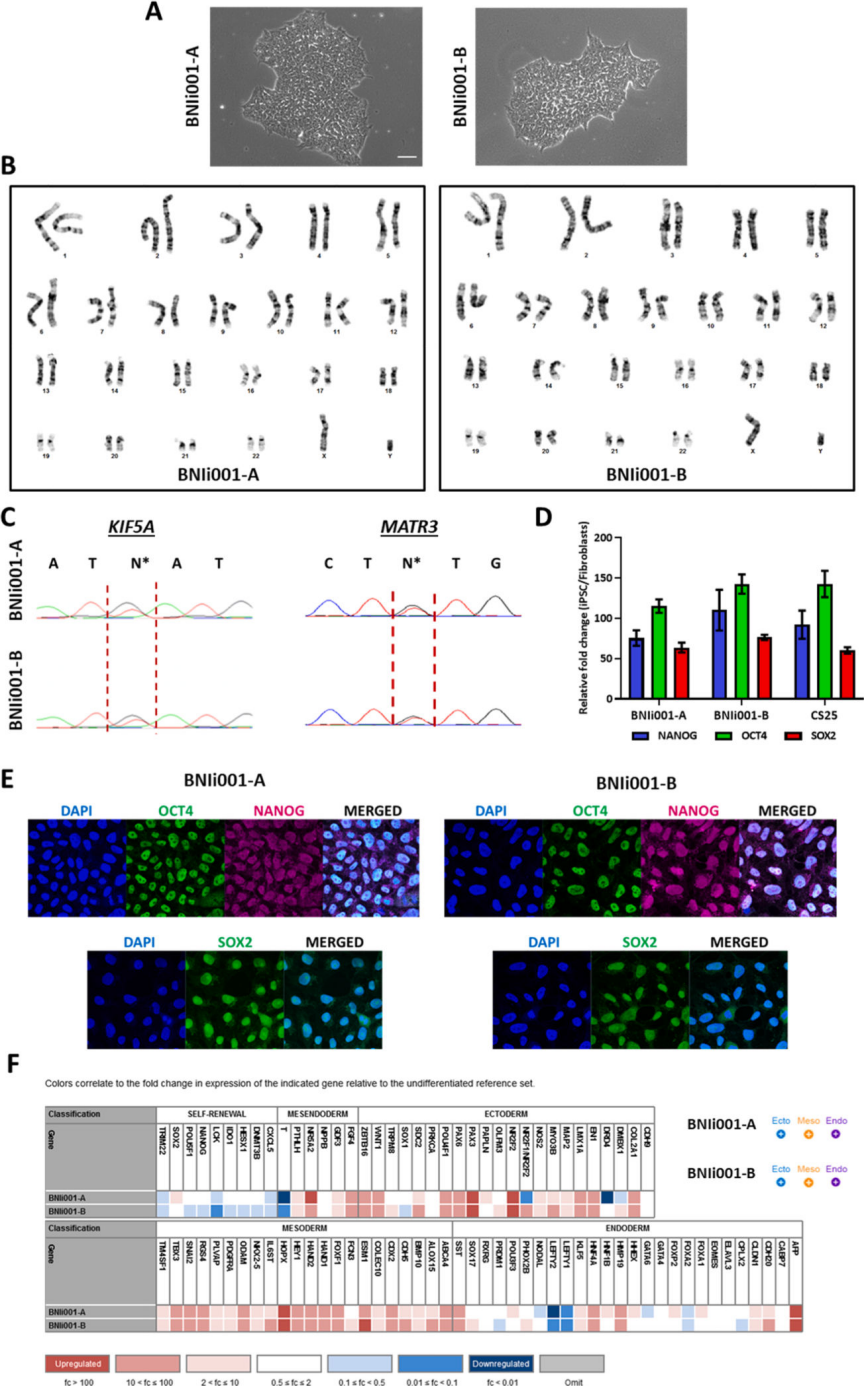
Characterization of iPSC lines BNIi001-A and BNIi001-B.

To generate the iPSCs, fibroblasts were isolated from a 50-year-old male ALS patient that carried the p.F115C mutation in MATR3. This patient served as the proband of family known as USALS#3 (Johnson et al., 2014). Subsequent to initial identification of the MATR3 mutation in this patient, further investigations also demonstrated the presence of an intronic mutation in KIF5A gene (Saez-Atienzar et al., 2020). Mutations in KIF5A had previously been identified as an ALS-causing gene (Brenner et al., 2018; Nicolas et al., 2018). Fibroblasts were converted to iPSC using Cytotune 2 Sendai Kit (Life Technologies) according to the manufacturer instructions. Resulting colonies were subsequently treated as separate clones, with clones 6 *(cell line BNIi001-A)* and 12 *(cell line BNIi001-B)* being chosen for further characterization because of their speed of growth and ability to form colonies (Fig. 1A). Karyotyping revealed there were no chromosomal abnormalities resulting from the reprogramming (Fig. 1B). Sequencing indicated retention of both mutations in the MATR3 and KIF5A genes (Fig. 1C) which were previously described (Johnson et al., 2014; Saez-Atienzar et al., 2020) in the patient. Further, RT-qPCR was used to demonstrated significantly elevated mRNA levels of pluripotency markers NANOG, OCT4, and SOX2 mRNA in iPSCs, including stem cell line CS25 which was obtained from the Cedar Saini IPSC core. Expression levels were compared to fibroblasts cells from the patient normalized to actin mRNA (Fig. 1D). Immunofluorescence was used to demonstrate protein expression of NANOG, OCT4, and SOX2 in iPSCs (Fig. 1E). To confirm the ability of iPSCs to generate all three germ layers, iPSCs were allowed to spontaneously differentiate in culture. Differentiation potential was assessed using the TaqMan hPSC Scorecard (Thermo Fisher) (Fig. 1F), and both clones demonstrated ability to differentiate into all 3 germ layers.

## 3. Materials and methods

*Brief summary of culture conditions, methodology used for cell line generation, genetic modification, and analyses performed. Describe in detail any new methodology used, summarise established protocols. Please include details of antibodies and primers separately in* Table 3*.*

### 3.1. Cell culture

Small pieces of a 2–3-mm forearm skin biopsy were plated on gelatin-coated tissue culture dishes. Fourteen to 28 days after initial plating, intense outgrowth of fibroblasts from the skin fragments were observed. Patient derived fibroblasts were grown in DMEM media with 10% FBS and Pen/Strep. Early (p2-p9) passages were used for reprogramming. iPS cell lines were generated by reprogramming fibroblasts with Cytotune 2 Sendai Kit from Life Technologies, using a variation of their recommended protocol as described in (Beers et al., 2015). iPSCs were plated and grown as a monolayer on Matrigel coated plates in MTeSR medium (Stem Cell Technologies) at 37 °C/5% CO_2_/95% humidity. Cells were passaged at 1:10 every 4–7 days.

### 3.2. Immunofluorescence assay

iPSCs were plated on coverslips, fixed in 4% paraformaldehyde for 5 mins, permeabilized using 0.03% v/v Triton-X in 1× Phosphate Buffered Saline (PBS). Cells were then blocked using Superblock at room temperature for 1 hr. Cells were incubated in primary antibodies at 4 °C overnight, washed, then incubated in secondary antibody for 1 h at room temperature. Coverslips were mounted with Vectashield with DAPI (Vector Laboratories) and an Observer Z1 (Zeiss) confocal microscope was used was used to image cells.

### 3.3. RNA extraction and qPCR

RNA for quantitative PCR was extracted from iPSC at passage 8 using the PureLink™ RNA mini kit (Invitrogen) following the manufacturer’s instructions. Following extraction, cDNA was created using SuperScript IV VILO Master Mix (Thermo Fisher Scientific) following the manufacturer’s instructions. Finally, qPCR was performed using the StepOnePlus RT-PCR (Applied Biosystems) machine. qPCR was performed with SYBR green (Applied Biosystems) conducted at 95 °C for 2 min, and then 40 cycles of 95 °C for 3 s and 60 °C for 30 s. Comparative ΔΔCt was analysed using the StepOne Software to calculate relative fold change.

### 3.4. Sequencing

Genomic DNA obtained from iPSCs, using the Wizard® SV Genomic DNA Purification System (Promega), was subject to PCR amplification using the primers shown in Table 3. The PCR product was then used as template for a standard sequencing reaction at the ASU Genomics Facility (MATR3) and at the Laboratory of Neurogenetics, NIA (KIF5A). Sanger sequencing was performed with capillary electrophoresis.

### 3.5. Karyotyping

Karyotyping of cells was performed by the Molecular Medicine Laboratory at St. Joseph’s Hospital and Medical Center (Phoenix, Arizona). Cells were analysed at passage 10, and 20 cells were assessed at a resolution of 400–425 bands per haploid set.

### 3.6. Mycoplasma test

Mycoplasma contamination was analysed with the LookOut® My-coplasma PCR Detection Kit.

## Supplementary Material

Supp.Figure1

## Figures and Tables

**Table 1.** Summary of lines.

iPSC line names	Abbreviation in figures	Gender	Age	Ethnicity	Genotype of locus	Disease
BNIi001-A BNIi001-B		Male	50	Caucasian		ALS

**Table 2:** Characterization and validation.

Classification	Test	Result	Data
Morphology	Brightfield Microscopy	Normal colony formation	Fig. 1A
Phenotype	Qualitative protein analysis Immunofluorescence	OCT4, NANOG, SOX2	Fig. 1E
	Quantitative mRNA analysis	OCT4, NANOG, SOX2	Fig. 1D
Genotype	Karyotype (G-banding) and resolution	46XY, Resolution 400–425	Fig. 1B
Identity	Sequencing	Demonstrated presence of both novel mutations described in Johnson et al 2014 and Saez-Atienzar et al. 2020	
Mutation analysis (IF APPLICABLE)	Sequencing	*Heterozygous mutations in MATR3 and KIF5A genes*	Fig. 1C
Microbiology and virology	Mycoplasma	*Mycoplasma testing by PCR*	Supplementary Fig. 1
Differentiation potential	Scorecard	*Result demonstrated ability of iPSCs to differentiate into all 3 germ layers*	Fig. 1F
Donor screening (OPTIONAL)	HIV 1 + 2 Hepatitis B, Hepatitis C	*NOT Done*	N/A
Genotype additional info (OPTIONAL)	Blood group genotyping	*Not Done*	N/A
	HLA tissue typing	*Not Done*	N/A

**Table 3.** Reagents details.

Antibodies used for immunocytochemistry			
	Antibody	Dilution	Company Cat # and RRID
Pluripotency Markers	NANOG	1:500	R&D systems Cat#AF1997
	OCT4	1:500	Stem Cell Technologies Cat#60093
	SOX2	1:500	Stem cell Technologies Cat#60055
Secondary antibodies	Donkey Anti-Goat	1:500	Invitrogen Cat# A-11056
	Goat Anti-Mouse	1:500	Invitrogen Cat# A-11001
**Primers**	**Target**	**Forward/Reverse primer (5′-3′)**	
Pluripotency Markers (qPCR)	*NANOG*	*CCTGTGATTTGTGGGCCTG/GACAGTCTCCGTGTGAGGCAT*	
	*OCT4*	*GGAAGGAATTGGGAACACAAAGG/AACTTCACCTTCCCTCCAACCA*	
	*SOX2*	*GCTACAGCATGATGCAGGACCA/TCTGCGAGCTGGTCATGGAGTT*	
Targeted mutation analysis	MATR3 Fwd	*GTCATTCCAGCAGTCATCTCTC*	
	MATR3 Rev	*TCCCTACACCTTTCTCCATCT*	
	KIF5A Ex27 Fwd	*AAGGGATTAAGATGGGAGAG*	
	KIF5A Ex27 Rev	*GGCACAAGGGAAATTTAATC*	

**Resource Table:** 

Unique stem cell lines identifier	**BNIi001-A** **BNIi001-B**
Alternative names of stem cell lines	
Institution	Barrow Neurological Institute; NIH\NHLBI iPS and Genome Engineering Core
Contact information of distributor	Robert Bowser, Robert.bowser@dignityhealth.org
Type of cell lines	iPSC
Origin	Human
Cell Source	Fibroblasts
Clonality	Clonal, clone #6 (BNIi001-A) & 12 (BNIi001-B)
Method of reprogramming	Sendai viral reprogramming
Multiline rationale	Isogenic clones
Gene modification	Yes
Type of modification	Spontaneous mutations (intronic mutation in KIF5A; missense mutation in MATR3)
Associated disease	Amyotrophic Lateral Sclerosis (ALS)
Gene/locus	**MATR3** p.Phe115Cys chr5:138643448,T > G **KIF5A**chr12:57582588G > T, NM_004984.3:c.2993-14G > T
Method of modification	N/A
Name of transgene or resistance	N/A
Inducible/constitutive system	N/A
Date archived/stock date	Date cell line archived or deposited in repository
Cell line repository/bank	N/A
