# Supplementary figures and images for: Generation of two induced pluripotent stem cell (iPSC) lines from an ALS patient with simultaneous mutations in KIF5A and MATR3 genes
Source: Stem Cell Res. Author manuscript; Available in PMC 2022 Jun 24. (PMC8222416; doi:10.1016/j.scr.2020.102141)

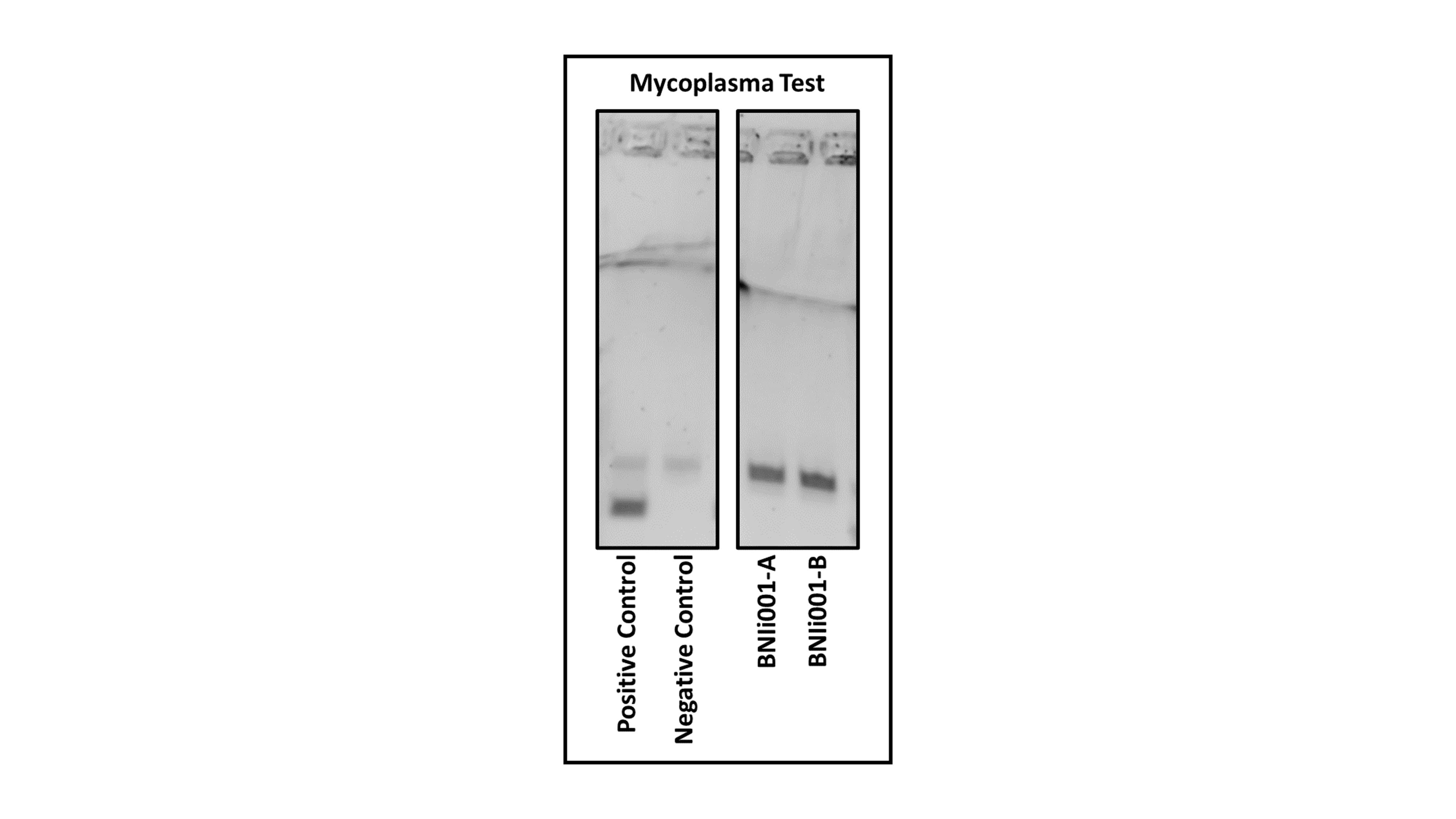

Supplement: Supp.Figure1 [file NIHMS1670727-supplement-Supp_Figure1.jpg]
